# The dietary inflammatory index and cardiometabolic parameters in US firefighters

**DOI:** 10.3389/fnut.2024.1382306

**Published:** 2024-06-13

**Authors:** Andria Christodoulou, Costas A. Christophi, Mercedes Sotos-Prieto, Steven Moffatt, Longgang Zhao, Stefanos N. Kales, James R. Hébert

**Affiliations:** ^1^Cyprus International Institute for Environmental and Public Health, Cyprus University of Technology, Limassol, Cyprus; ^2^Department of Environmental Health, Harvard T.H. Chan School of Public Health, Boston, MA, United States; ^3^Department of Preventive Medicine and Public Health, School of Medicine, Universidad Autónoma de Madrid, Madrid, Spain; ^4^Biomedical Research Network Centre of Epidemiology and Public Health (CIBERESP), Carlos III Health Institute, Madrid, Spain; ^5^IMDEA-Food Institute, CEI UAM + CSIC, Madrid, Spain; ^6^National Institute for Public Safety Health, Indianapolis, IN, United States; ^7^Department of Epidemiology and Biostatistics, Arnold School of Public Health, University of South Carolina, Columbia, SC, United States; ^8^Cancer Prevention and Control Program, Arnold School of Public Health, University of South Carolina, Columbia, SC, United States; ^9^Connecting Health Innovations LLC, Columbia, SC, United States; ^10^Department of Occupational Medicine, Cambridge Health Alliance and Harvard Medical School, Cambridge, MA, United States

**Keywords:** DII scores, cardiovascular disease, US firefighters, nutrition, inflammatory index

## Abstract

**Introduction:**

Dietary choices play a crucial role in influencing systemic inflammation and the eventual development of cardiovascular diseases (CVD). The Dietary Inflammatory Index (DII®) is a novel tool designed to assess the inflammatory potential of one’s diet. Firefighting, which is characterized by high-stress environments and elevated CVD risk, represents an interesting context for exploring the dietary inflammatory-CVD connection.

**Aim:**

This study aims to investigate the associations between Energy-adjusted Dietary Inflammatory Index (E-DII™) scores and cardiometabolic risk parameters among US firefighters.

**Methods:**

The study analyzed 413 participants from the Indianapolis Fire Department who took part in a Federal Emergency Management Agency (FEMA)-sponsored Mediterranean diet intervention trial. Thorough medical evaluations, encompassing physical examinations, standard laboratory tests, resting electrocardiograms, and submaximal treadmill exercise testing, were carried out. Participants also completed a detailed food frequency questionnaire to evaluate dietary patterns, and E-DII scores were subsequently computed based on the gathered information.

**Results:**

Participants had a mean body mass index (BMI) of 30.0 ± 4.5 kg/m^2^ and an average body fat percentage of 28.1 ± 6.6%. Regression analyses, adjusted for sex, BMI, maximal oxygen consumption (VO_2_ max), max metabolic equivalents (METS), age, and body fat percentage, revealed significant associations between high vs. low E-DII scores and total cholesterol (*β* = 10.37, *p* = 0.04). When comparing low *Vs* median E-DII scores there is an increase in glucose (*β* = 0.91, *p* = 0.72) and total cholesterol (*β* = 5.51, *p* = 0.26).

**Conclusion:**

Our findings support an association between higher E-DII scores and increasing adiposity, as well as worse lipid profiles.

## Introduction

Cardiovascular diseases (CVD) account for more than 30% of global deaths, with more than 18 million people dying from CVD-related conditions (1). Several studies have identified inflammation as the main underlying cause of CVD (2, 3). A poor diet, which includes large quantities of calorically dense foods, rich in simple carbohydrates and fat, is known to be pro-inflammatory (4, 5). The chronic inflammation caused by this diet provides a substrate for mechanisms that operate systemically or locally through tissue-simmering inflammation to increase the risk of CVD (6, 7). By contrast, a nutrient-dense diet abundant in fruits, vegetables, and fish exhibits anti-inflammatory properties that can improve immune response, thereby offering protection against a range of chronic non-communicable diseases, including diabetes, cancer, and CVD (8, 9) as well as infectious diseases such as COVID-19 (10, 11).

The Dietary Inflammatory Index (DII®) was developed to quantify the potential of diet in connection to putative inflammation-related health parameters (12). The DII quantifies the impact of diet on an individual’s potential level of inflammation, based on 45 food parameters. The DII was developed and validated by researchers at the University of South Carolina and has been used extensively in different populations and across a wide variety of health parameters (13, 14). The Energy-adjusted Dietary Inflammatory Index (E-DII™) was developed to account for the influence of total energy consumption on inflammation. Both the DII and E-DII are based on the careful review and as in scoring of 1943 peer-reviewed articles (14). A pro-inflammatory diet, indicated by a high DII score, typically contains high concentrations of saturated fats and refined carbohydrates, and low concentrations of polyunsaturated fatty acids (PUFAs) and flavonoids. A low DII score results from consuming less energy-dense foods and more fruits, and vegetables. Evidence has shown that the DII can capture the inflammatory potential from diet and, therefore, represents an important innovation to address the association between dietary inflammation and health parameters (15, 16). Over 50 studies have validated the use of the DII or E-DII as a measure of inflammation and linked it to markers such as CRP, IL-6, and TNF-α- R2 (17–21). Additionally, over 1,000 studies demonstrated an increased risk of chronic disease, including CVD and metabolic syndrome, in people with higher DII scores (22–25).

The most frequent cause of on-duty death among firefighters is sudden cardiac death (SCD), due to underlying CVD and/or cardiomegaly (26), with almost half of all on-duty fatalities attributed to CVD (27, 28) and caused by SCD, strokes, aneurysms, and other CVD-related conditions (22, 23). Firefighters have very demanding jobs and food habits that are generally consistent with those of the general US population despite the strenuous nature of their work, coupled with irregular shift schedules: many of their meals at the fire station consist of processed foods, including sugar-sweetened beverages (29–31).

There have not been many studies looking at the association of DII and cardiovascular risk parameters among firefighters. A study conducted by Vatandoost et al., among firefighters of the Tehran region in Iran, suggested that HDL and hs-CRP levels are high among firefighters with high DII scores. However, there were no significant differences in the means of HDL cholesterol and CRP levels in the observed population, after adjusting for other relevant confounders (32).

Considering the importance of firefighters being in good physical condition, and the demanding nature of their work, it is important to investigate the association between diet-associated inflammation and CVD risk indices in firefighters. The aim of this study is to identify any association between DII/E-DII scores and cardiometabolic parameters among US firefighters. This would give us a better understanding of the dietary habits of firefighters and provide a connection of the E-DII scores (derived from the dietary habits of this population) and use them to evaluate whether those with high EDII levels have further risk for CVD, compared to those with low E-DII scores.

## Materials and methods

### Study participants

Firefighters were recruited between November 2016 and April 2018 to participate in the study “Feeding America’s Bravest: Mediterranean Diet-Based Interventions to change firefighters’ Eating Habits and Improve Cardiovascular Risk Profiles.” More details on the study methodology and participant recruitment are available elsewhere (33). All data used in the current analysis are from baseline measurements.

### Dietary assessment and diet inflammatory index

A validated food frequency questionnaire was administered prior to the commencement of the study to establish baseline dietary habits among participants. The questionnaire collected information on the food consumption of 131 different items (as an average consumption over the period of the previous year). Food items included fruit, vegetables, meat, cereal, sweets, baked goods etc. (33). Participants in each survey were questioned about their frequency of consuming specific foods over the past year. For each food item, a standard portion size and nine response options ranging from “never or less than once per month” to “6 or more times per day” were provided. Nutrient and energy intake was determined by multiplying the reported food consumption with its respective nutrient and energy content using data from the US Department of Agriculture database, supplemented with information from manufacturers. The validation of the questionnaire is described in detail elsewhere (34).

The inflammatory potential of individuals’ diets was assessed using the Dietary Inflammatory Index(DII)and the Energy-adjusted DII E-DII. These indices measure the inflammatory impact of diets on a scale ranging from highly anti-inflammatory (most negative score) to highly pro-inflammatory (most positive score). The DII was developed through a comprehensive process, detailed elsewhere (35), involving a thorough literature review that identified 45 food parameters, such as macronutrients, vitamins, minerals, flavonoids and whole food items linked to six inflammatory biomarkers [interleukins (IL)-1b, −4, −6, −10, tumor-necrosis factor-alpha (TNFα), and C-reactive protein (CRP)]. From the information collected from the FFQ, 29 food parameters were available for further use.

To calculate DII scores, these self-reported values were converted into *z*-scores using a global comparative database from 11 countries, by subtracting from the individual’s self-report value the mean of the global database then dividing by the standard deviation (14, 36). *Z*-scores were then transformed into proportions (ranging from 0 to 1), centered on zero by doubling each and subtracting 1. The sum of these scores provided the overall DII score. Energy-adjusted DII (E-DII™) scores, calculated per 1,000 kcal consumption using a density approach, followed a similar procedure but utilized an energy-adjusted global comparison database (2, 3).

Both DII and E-DII scores can range from approximately −9 to +8; indicating a spectrum from minimally to maximally pro-inflammatory, respectively. The scoring methodology and scaling are consistent between DII and E-DII, ensuring compatibility across different studies (2).

For this study, all available food parameters from the FFQ (29 of 45) were used to calculate an individual’s overall DII score as shown in Appendix 1 (37). For the E-DII, energy was in the denominator; so, 28 parameters were used for computation. The food parameters used included: alcohol, caffeine, zinc, fiber, omega-3, Isoflavones, selenium, flavonols, Vitamin B12, carbohydrate, anthocyanidins, iron, Omega-6, protein, selenium, flavonones, Vitamin B6, cholesterol, magnesium, riboflavin, tjiamin, beta carotene, Vitamin D, fat, niacin, flavones, SFA, Vitamin C and energy.

### Anthropometric parameters

Data on physical activity was collected during the participants’ assessments from the fire department medical examinations at Public Safety Medical (PSM) clinics, which is led by an IFD-contracted physician. The examinations included the collection of occupational, smoking, and medical history; a physical examination, including body mass index (BMI = weight (kg)/height (m)^2^) and body fat measurements (using bioelectrical impedance); routine laboratory tests; resting electrocardiograms; and maximal treadmill exercise testing.

### Risk parameters

During the initial visit, participants underwent assessments of blood pressure and anthropometrics. Resting blood pressure was measured using an appropriately sized cuff while participants were seated. Height and weight were recorded, and Body Mass Index in kg/m^2^ was calculated for all of the study participants. Additionally, the percentage of body fat was estimated using a Bioelectrical Impedance Analyzer (BIA) (38, 39).

Other biochemical indices were evaluated during medical examinations. Measurements were taken closest to the date of study consent and within the same 12-month period. Blood samples were collected after an overnight fast using ethylenediaminetetraacetic acid (EDTA) collection tubes, with 15 mL of blood collected. Plasma was frozen at –80°C, and blood lipid profiles were determined using an automated high-throughput enzymatic analysis. The coefficient of variation values for this analysis were ≤3% for cholesterol and ≤5% for triglycerides, utilizing the cholesterol assay kit and reagent (Ref: 7D62-21) and triglycerides assay kit and reagent (Ref: 7D74-21) by the ARCHITECT c System, Abbott Laboratories, Abbott Park, IL, USA.

### Statistical analysis

Characteristics measured on a continuous scale and having a normal distribution are presented as mean ± standard deviation (SD), whereas categorical variables are reported as frequency (percentage). Model Goodness of Fit statistics indicated that the E-DII performed better than the DII. Values are shown stratified by levels of E-DII score (median vs. low, with cut-off point being the median = −0.09) and compared using the t-test and the chi-square test of independence, for quantitative and qualitative characteristics, respectively. Linear regression models were fit to assess the effect of E-DII score on cardiometabolic risk, after adjusting for age, gender, BMI, body fat percent, max metabolic equivalents (METS), and oxygen consumption (VO_2_). The resulting beta coefficients are presented, together with the corresponding 95% confidence intervals and *p*-values. As a sensitivity analysis, E-DII scores were used in the models as continuous variables as well as in a tertiles form (low vs. median). All the statistical analyses were performed using SAS version 9.4 (SAS Institute Inc., Cary, NC, USA). The alpha level of significance was set at 0.05 and all the tests performed were two-sided.

## Results

There were 413 firefighters who participated in the study. Participants were primarily male (94%) with the average age being 48 years old. The mean weight of the participants was 97 kg and the median E-DII was 0.085. Individuals with E-DII scores above the median had higher BMI (30.6 kg/m^2^ ± 4.7), body fat percentage (28.8 ± 0.4), and triglycerides (145.8 mg/dL ± 156.3), compared to those whose E-DII scores were below the median (Table 1). Total cholesterol, HDL cholesterol, and cholesterol ratio were also significantly associated with higher E-DII scores (200.9 mg/dL ± 38.5, 47.9 mg/dL ± 11.1, and 4.41 ± 0.10, in the high E-DII group, respectively). LDL cholesterol (123.6 mg/dL ± 35.9) and glucose (100.6 mg/dL ± 19.8) were also higher in the participants with high E-DII, however, those differences were not statistically significant.

**Table 1:** Baseline characteristics.

Characteristic		Overall (*n* = 413)	E- DII Score		
			Low (*n* = 213)	High (*n* = 213)	*p*
Sex	Male	390 (94%)	193(46%)	201(48)	
	Female	23(6%)	16(4%)	7(2%)	0.055
Age (years)		48.4 ± 8.2	45.6 ± 8.7	44.6 ± 7.8	0.216
Height(m)		1.77 ± 0.07	1.78 ± 0.07	1.79 ± 0.06	0.102
Weight(kg)		96.8 ± 17.3	92.6 ± 17.5	97.0 ± 18.6	0.014
Diastolic BP(mmHg)		78.4 ± 6.1	78.3 ± 6.0	81.03 ± 28.4	0.134
Systolic BP(mmHg)		123.3 ± 8.9	181.8 ± 17.1	180 ± 22.5	0.356
BMI (kg/m^2^)		30.0 ± 4.5	29.3 ± 4.1	30.6 ± 4.7	0.002
% body fat		28.1 ± 6.1	27.3 ± 0.5	28.8 ± 0.4	0.03
Max METS*		11.5 ± 5.4	11.4 ± 2.2	11.6 ± 7.3	0.78
VO_2_ max		42.1 ± 4.9	42.3 ± 5.3	41.7 ± 4.6	0.22
Total cholesterol (mg/dl)		196.9 ± 38.3	192.8 ± 37.8	200.9 ± 38.5	0.03
HDL cholesterol (mg/dl)		49.1 ± 11.4	50.4 ± 11.6	47.9 ± 11.1	0.02
LDL cholesterol (mg/dl)		121.9 ± 34.6	120.2 ± 33.2	123.6 ± 35.9	0.33
Cholesterol ratio		4.20 ± 1.30	3.98 ± 0.07	4.41 ± 0.10	0.001
Triglycerides (mg/dl)		129.3 ± 117.1	113.3 ± 60.7	145.8 ± 156.3	0.005
Glucose (mg/dl)		100.0 ± 20.3	99.3 ± 20.9	100.6 ± 19.8	0.50

*Metabolic equivalents - one MET is defined as 3.5mL O2 uptake/Kg per min, which is the resting oxygen uptake in a sitting position.

The association between E-DII scores and different cardio-metabolic parameters is shown in Table 2. After adjusting for age, gender, VO_2_ max, max METS, BMI, and body fat %, higher levels of E-DII were significantly associated with elevated cholesterol (*β* = 10.37, *p* = 0.04) and cholesterol ratio (*β* = 0.32, *p* = 0.05), when comparing high to low scores of E-DII. We also observed an increase in LDL cholesterol (*β* = 6.51, *p* = 0.14) and triglycerides (β = 23.9, *p* = 0.11), though these effects were not statistically significant. When comparing low with median E-DII scores there is an increase in glucose (*β* = 0.91, *p* = 0.72) and total cholesterol (*β* = 5.51, *p* = 0.26), however these results were not significant.

**Table 2:** Association of E-DII scores with cardiometabolic parameters.

Outcome	High vs. Low E-DII			Low vs. High EDII		
	*β*	95% CI	*p*	*β*	95% CI	*p*
BMI	−0.13	−1.14, 0.89	0.81	0.49	−0.54,1.49	0.36
Body Fat%	−0,40	−1.81, 1.02	0.58	1.13	−0.29, 2.54	0.12
Total cholesterol	5.51	−4.02, 15.04	0.26	10.37	0.74, 21.01	0.04
HDL cholesterol	−1.38	−4.09, 1.32	0.32	−1.02	−3.75, 1.71	0.46
LDL cholesterol	7.85	−0.80, 16.49	0.08	6.51	−2.21, 15.22	0.14
Cholesterol ratio	0.20	−0.12, 0.52	0.22	0.32	−0.00, 0.65	0.05
Triglycerides	−3.96	−33.0, 25.1	0.79	23.9	−5.48, 53.30	0.11
Glucose	0.91	−4.11, 5.93	0.72	0.15	−4.93, 5.23	0.95

*Model adjusted for age, gender, VO2 max, max METS; model additionally adjusted for BMI for all cardiometabolic parameters except for BMI and body fat (%).

## Discussion

We observed that a large proportion of firefighters in our study have a high inflammatory diet, something that is expected, as we had previously observed that most of our study population follows a Standard American Diet, which is characterized by high consumption of meat and processed food (40). This observation is in agreement with previous studies that also conducted an in-depth investigation on the dietary habits of firefighters (41–44).

In our study, we observed an association between E-DII scores and negative cardiometabolic risk. This aligns with the findings presented by Ruiz-Canela et al., indicating that an elevated DII correlated with increased general obesity and abdominal obesity levels, while accounting for the impact of adherence to a Mediterranean diet on inflammation (45). E-DII was associated with higher BMI (*β* = 0.49, *p* = 0.36), cholesterol ratio (*β* = 0.32, *p* = 0.05) and total cholesterol (*β* = 10.37, *p* = 0.04). This is in agreement with the findings of Sokol et al. (46), who also studied the association of individuals with high DII scores and their increased risk of obesity, high triglyceride levels, and an increased waist-to-hip ratio. Different associations with E-DII exist due to variations across study populations and individuals who may respond differently to different diet interventions. To some extent, these associations between diet and cardiometabolic parameters can be influenced by genetic factors as well as environmental factors. In addition, different studies may differ methodologically and these can also lead to differences in the observed association between E-DII and cardiometabolic risk.

Our findings also corroborate several recent research studies highlighting the association between DII and cardiometabolic conditions (47–50). Moreover, studies have shown that a diet with a high DII score promotes systematic inflammation which, in turn, contributes to the development of CVD (51).

We also found that higher E-DII scores were significantly associated with a higher BMI and %body fat of the participants. Other studies have also reported that overweight and obese individuals have higher levels of systemic inflammation and greater risk for CVD. These findings could be affected by the different characteristics of the participants, like age, gender, and ethnicity. For example, studies involving mostly Caucasian individuals showed a greater association between DII scores and cardiovascular disease (32).

We also observed elevated levels of cholesterol in participants with higher E-DII scores. This was true even after adjusting for gender, BMI and %body fat. A study among Indonesian males also demonstrated an increase in cholesterol levels as DII scores increase (52). High cholesterol levels can be considered a “silent killer,” as an increase in cholesterol is associated with high risk for atherosclerosis (53, 54).

The strengths of our study include the relatively large number of firefighters, whose records were collected by medical professionals and these records were available throughout the study. Additionally, the FFQ used was very extensive and was designed to capture different eating habits within the USA. The FFQ provided 29 food parameters that were used in the calculation of the E-DII scores. Despite its strengths, our study had some limitations. This included the use of only baseline dietary information, which was self-reported. This limitation is common in dietary studies highlighting the need to use more robust dietary assessment methods in the future. However, good options are not readily available at reasonable cost. Also, we are reliant on self-reports of diet, which are subject to a variety of information biases. Even though previous research has shown that a drop-off from the maximum 45 parameters to 28 parameters does not materially change the risk estimate for calculating E-DII scores, there were only 29 food parameters in our analysis (37). Despite these weaknesses, our findings contribute valuable insight information on the association of inflammation, diet, and cardiometabolic risk. This could provide the foundation by which more targeted interventions are designed to accommodate the unique health challenges faced by firefighters and thus, ultimately, will enable them to improve their overall health.

## Conclusion

In conclusion, our study was able to assess the relationship between E-DII scores and cardiometabolic parameters among firefighters. The results not only add to the current knowledge, but they also improve our understanding by showing an increased risk of CVD even within the demographic of US firefighters. Further research is needed to determine the link between diet, inflammation, and cardiovascular disease among firefighters, in order to help design better strategies improving the well-being of these individuals.

## Data availability statement

The raw data supporting the conclusions of this article will be made available by the authors, without undue reservation.

## Ethics statement

The studies involving humans were approved by The study was conducted in accordance with the Declaration of Helsinki, and approved by the Institutional Review Board of Harvard Institutional Review Board (IRB16-10170) and is registered at Clinical Trials (NCT029441757). The studies were conducted in accordance with the local legislation and institutional requirements. The participants provided their written informed consent to participate in this study.

## Author contributions

AC: Writing – original draft, Writing – review & editing. CC: Conceptualization, Data curation, Formal analysis, Methodology, Supervision, Writing – review & editing. MS-P: Conceptualization, Methodology, Resources, Writing – review & editing. SM: Project administration, Writing – review & editing. LZ: Resources, Writing – review & editing. SK: Conceptualization, Funding acquisition, Methodology, Project administration, Supervision, Writing – review & editing. JH: Resources, Supervision, Writing – review & editing.

## Appendix 1

**Table:**

Food parameters			
Alcohol	Vitamin B12	Vitamin B6	Beta Carotene
Caffeine	Carbohydrate	Cholesterol	Vitamin D
Zinc	Anthocyanidins	Energy	Fat
Fiber	Iron	Magnesium	Niacin
Omega 3	Omega 6		Flavones
Isoflavones	Protein	Riboflavin	SFA
Selenium	Selenium	Thiamin	Vitamin C
Flavanols	Flavonones		
